# Whole Genome Sequencing of 39 Invasive *Streptococcus pneumoniae* Sequence Type 199 Isolates Revealed Switches from Serotype 19A to 15B

**DOI:** 10.1371/journal.pone.0169370

**Published:** 2017-01-03

**Authors:** Oliwia Makarewicz, Marie Lucas, Christian Brandt, Leonie Herrmann, Andreas Albersmeier, Christian Rückert, Jochen Blom, Alexander Goesmann, Mark van der Linden, Jörn Kalinowski, Mathias W. Pletz

**Affiliations:** 1 Center for Infectious Diseases and Infection Control, Jena University Hospital, Jena, Germany; 2 Center for Biotechology, University of Bielefeld, Bielefeld, Germany; 3 Institute for Bioinformatics and Systems Biology, Justus Liebig University Giessen, Giessen, Germany; 4 German National Reference Center of Streptococci, University Hospital RWTH Aachen, Aachen, Germany; Instituto Butantan, BRAZIL

## Abstract

*Streptococcus pneumoniae* is a major pathogen that causes different invasive pneumococcal diseases (IPD). The pneumococcal polysaccharide capsule is a main virulence factor. More than 94 capsule types have been described, but only a limited number of capsule types accounted for the majority of IPD cases before the introduction of pneumococcal vaccines. After the introduction of the conjugated pneumococcal vaccine PCV7, which covered the seven most frequent serotypes in IPD in the USA, an increase in IPD caused by non-vaccine serotypes was observed, and serotype 19A, which belongs to sequence type (ST) 199, was among the most prevalent STs. After the introduction of the extended vaccine PCV13, which includes serotype 19A, serogroup 15B/C increased in IPD. Therefore, whole genome sequences of 39 isolates of ST199 from Germany (collected between 1998 and 2011) with serotype 19A (n = 24) and serogroup 15B/C (n = 15) were obtained using an Illumina platform and were analysed to identify capsular switches within ST199. Two 19A to 15B/C serotype switch events were identified. Both events occurred before the introduction of PCV7, which indicates that a capsular switch from 19A to 15B among ST199 isolates is not unusual and is not directly linked to the vaccination. The observed serotype replacement appears to be the result of a vacant niche due to the displacement of vaccine serotypes that is now successfully occupied by ST199 clones.

## Introduction

*Streptococcus pneumoniae* are encapsulated, facultative anaerobic, non-sporulating Gram-positive bacteria that usually occur as diplococci. *S*. *pneumoniae*, also known as pneumococcus, is a major pathogen that causes community-acquired pneumonia (CAP), acute exacerbations of chronic bronchitis, meningitis, sinusitis, otitis media, and sepsis. Pneumococcal infections usually involve infants, immunocompromised individuals and the elderly. Non-invasive diseases (i.e., sinusitis and otitis media) are frequent but not severe. Invasive pneumococcal diseases (IPD) refer to the isolation of *S*. *pneumoniae* from a normally sterile site, e.g., blood, cerebrospinal fluid, or pleural fluid, and have a lower incidence but are associated with a high case fatality rate [1]. The main reservoir of *S*. *pneumoniae* is the nasopharyngeal zone of healthy carriers, particularly infants. Up to 70% of infants attending day care centres and more than 90% of infants in some native communities [2], but less than 5% of immune-competent adults, are colonized [2–4]. However, in HIV-endemic regions, the prevalence among the parents might be significantly higher, with rates of 43.2% in HIV-infected parents and 26.8% in HIV-non-infected parents [5].

The genome of *S*. *pneumoniae* is relatively small and highly variable. Molecular epidemiology studies using multi-locus sequence typing (MLST) have classified 11,176 different pneumococcal sequence types (ST) that are subordinate to approximately 100 clonal complexes (CC) (http://pubmlst.org/spneumoniae/, accessed 2 March 2016). The polysaccharide capsule, the synthesis of which is encoded by the *cps* locus, is a major virulence factor. This capsule is poorly recognized by phagocytes and protects pneumococci from phagocytosis. Pneumococci can be discriminated into different serotypes according to their different capsule types using the Neufeld Quellung reaction [6]. To date, more than 94 pneumococcal polysaccharide capsule types have been described, but only a limited number of serotypes account for the majority of infections, some of which are associated with antibiotic resistance.

The introduction of the conjugated pneumococcal vaccine PCV7, which covers the seven most frequent serotypes (4, 6B, 9V, 14, 18C, 19F and 23F), for use in infants between 2000 (USA) and 2006 (Germany) led to a substantial decrease in vaccine-serotype associated IPD and in non-vaccinated children and adults *via* herd protection effects [7, 8]. Simultaneously, increasing rates of IPD caused by the non-PCV7 serotypes were reported in different regions. Globally, the most important PCV7 replacement serotype was 19A, which was also associated with penicillin and macrolide resistance [9–14]. Molecular analyses revealed that a substantial proportion of those serotype 19A isolates belong to ST199 [11, 12, 15].

To account for the observed replacement, a 13-valent vaccine (PCV13) was introduced in 2009 in Germany and in 2010 in the USA. In addition to the seven serotypes in PCV7, this vaccine contains the most frequent replacement serotypes [16, 17] and serotypes with high prevalence in the developing world [5, 18] (1, 3, 5, 7F, 6A and 19A), which has led to an additional 94% reduction in IPD caused by those 13 vaccine serotypes [7, 19]. Currently, increased rates of IPD by serogroup 15B/C, which is predominantly associated with ST199, can be observed in some countries [20]. Therefore, 39 IPD isolates of ST199 and serotypes 19A, 15B and 15C collected in Germany between 1998 and 2011 were sequenced to investigate whether the observed spread of ST199 can be explained by a successful clone that switched its serotype and whether this switch is related to selective pressure from the introduced vaccinations.

## Results and Discussion

### Draft genome details

The sequencing project included all available isolates with ST199, serotype 15B (n = 9) and serotype 15C (n = 6) and 24 randomly chosen isolates of serotype 19A collected by the German National Reference Centre for Streptococci (Aachen, Germany) (GNRCS) in Germany. The whole-genome sequence libraries were constructed using a transpososome-mediated fragmentation technique and were sequenced in both directions with at least 35-fold coverage for 37 isolates and a 21- or 30-fold coverage for the two remaining isolates. On average, 50% of the draft genomes were covered by at least 75±12 kb in the contigs and 130±12 kb in the scaffolds, resulting in 65±18-fold coverage of the draft genomes. All draft genomes were deposited in GenBank under the accession numbers listed in Table 1.

**Table 1:** Characterization of the *S*. *pneumoniae* isolates used in this study. Resistance and susceptibility were determined based on EUCAST rules. MICs categorized as resistance^#^ are indicated in bold letters.

ID	serotype	Accession No.	Date of Sampling	Type of Infection	Sampling Material	Age in years	Age in months	MIC PCN	MIC CTX	MIC CLR	MIC CLI	MIC LVX	MIC TET	MIC VAN
13827	15B	FLUW01000001-FLUW01000068	04/1999	Pneumonia	Tracheal secretion	76		0.015	0.008	0.125	0.03	1	0.25	0.5
1541	15B	FLMH01000001-FLMH01000070	06/2003	Meningitis	CSF		36	0.015	0.015	0.125	0.06	1	0.25	0.25
20007	15B	FLMN01000001-FLMN01000059	03/2004	Pneumonia	Puncture		8	0.015	0.03	0.125	0.12	1	0.25	2
21299	15B	FLNA01000001-FLNA01000060	10/2004	Pneumonia	Blood	67		0.015	0.015	0.125	0.06	1	0.5	0.25
27401	15B	FLNL01000001-FLNL01000064	02/2006	Meningitis	CSF	11		0.015	0.03	0.125	0.12	1	0.12	2
28838	15B	FLSY01000001-FLSY01000068	10/2006	Meningitis	CSF	6		0.015	0.015	**4**	0.12	1	0.25	0.5
38301	15B	FLTA01000001-FLTA01000062	02/2009	Carriage	Nasopharynx		3	0.015	0.03	0.125	0.12	1	0.25	0.5
41023	15B	FLNI01000001-FLNI01000059	09/2009	Carriage	Nasopharynx		11	0.015	0.015	0.125	0.12	1	0.5	0.5
41844	15B	FLNG01000001-FLNG01000070	12/2009	Carriage	Nasopharynx		13	0.015	0.03	0.125	0.12	1	0.25	0.5
27516	15C	FLUC01000001-FLUC01000075	01/2005	Pneumonia	Sputum			0.015	0.015	0.125	0.12	0.5	0.5	2
23543	15C	FLMZ01000001-FLMZ01000051	08/2005	Sepsis	Blood		11	0.015	0.015	0.125	0.12	1	0.25	2
27700	15C	FLSW01000001-FLSW01000058	04/2006	Sepsis	Blood		36	0.015	0.03	0.125	0.12	1	0.25	2
40086	15C	FLSX01000001-FLSX01000061	05/2009	Carriage	Nasopharynx		2	0.015	0.03	0.125	0.12	1	1	0.5
37666	15C	FLNC01000001-FLNC01000060	10/2009	Carriage	Nasopharynx		11	0.015	0.03	0.125	0.12	1	0.5	0.5
44233	15C	FLUD01000001-FLUD01000058	04/2010	Otitis media	Middle ear fluid		29	0.015	0.015	0.5	0.12	1	0.25	0.5
**246**	19A	FLMI01000001-FLMI01000058	02/1998		Blood		19	0.06	0.03	0.125	0.06	0.5	0.25	0.12
376	19A	FLMJ01000001-FLMJ01000061	10/1998	Meningitis	CSF		6	0.015	0.015	0.03	0.06	1	0.25	0.25
13514	19A	FLMK01000001-FLMK01000063	02/1999	Sinusitis	Nasopharynx	5		**0.12**	0.03	0.03	0.03	1	0.12	2
762	19A	FLML01000001-FLML01000063	10/2000		Blood		14	0.015	0.015	0.125	0.06	1	0.5	0.5
893	19A	FLUU01000001-FLUU01000055	02/2001		Blood		8	0.015	0.015	0.125	0.06	0.5	0.5	0.5
20605	19A	FLNM01000001-FLNM01000060	06/2003	Pneumonia	Blood	81		0.015	0.015	0.125	0.06	1	0.25	0.25
1720	19A	FLMM01000001-FLMM01000057	02/2004	Meningitis	CSF	7		0.06	0.25	0.125	0.12	1	0.5	2
20253	19A	FLMO01000001-FLMO01000060	03/2004		Blood		12	0.015	0.03	0.125	0.12	1	0.25	2
21295	19A	FLMY01000001-FLMY01000060	06/2004	Pneumonia	Blood	82		0.015	0.015	0.125	0.06	0.5	0.25	0.25
23150	19A	FLUJ01000001-FLUJ01000081	05/2005	Meningitis, Sepsis	n.s.		21	**0.125**	0.03	**4**	0.12	0.5	0.5	2
36180	19A	FLUE01000001-FLUE01000061	11/2008	Otitis media	Middle ear fluid		17	**0.125**	0.06	0.125	0.12	1	0.5	1
37119	19A	FLNJ01000001-FLNJ01000058	12/2008	Otitis media	Middle ear fluid		10	0.015	0.03	0.125	0.12	**2**	0.5	0.5
44017	19A	FLNE01000001-FLNE01000057	04/2009	Pneumonia	Blood			**0.125**	0.03	0.125	0.12	1	0.5	0.5
37752	19A	FLNK01000001-FLNK01000068	09/2009	Mastoiditis	Ear swab		10	0.015	0.015	0.125	0.12	1	0.25	0.5
41617	19A	FLNF01000001-FLNF01000060	11/2009	Meningitis	CSF		35	**0.125**	0.03	0.125	0.12	1	0.25	0.5
42538	19A	FLSZ01000001-FLSZ01000058	01/2010	Sinusitis	Wound swab		13	0.03	0.06	0.125	0.12	**2**	0.25	0.5
42694	19A	FLUI01000001-FLUI01000067	02/2010	Otitis media	Middle ear fluid		11	**0.25**	0.06	**4**	0.12	1	0.5	0.5
43003	19A	FLNH01000001-FLNH01000070	02/2010	Pneumonia	Blood		17	0.015	0.015	0.125	0.12	1	0.5	0.5
43985	19A	FLMW01000001-FLMW01000066	03/2010	Meningitis	CSF		19	0.015	0.03	0.125	0.12	1	0.5	0.5
46048	19A	FLUG01000001-FLUG01000065	07/2010		Blood		22	0.015	0.03	0.125	0.12	1	0.5	0.5
46770	19A	FLNB01000001-FLNB01000056	10/2010		Blood		20	0.015	0.03	0.125	0.12	1	0.5	0.5
46464	19A	FLUF01000001-FLUF01000058	10/2010	Sepsis	Blood	14		0.015	0.015	0.125	0.12	1	0.25	0.5
47301	19A	FLUH01000001-FLUH01000067	12/2010	Meningitis	CSF	7		0.015	0.015	0.125	0.12	1	0.25	0.5
48565	19A	FLND01000001-FLND01000067	03/2011	Otitis media	Middle ear fluid		20	0.015	0.015	0.125	0.12	1	0.25	0.5

MIC—minimum inhibitory concentration in mg/L, ^#^ according to EUCAST, the low-level MIC of ≥ 0.125 mg/L penicillin is interpreted as nonsusceptible when meningitis is the focus of infection, OXA—oxacillin, PCN—penicillin, CTX—cefotaxime, CLR—clarithromycin, CLA—clindamycin, LVX—levofloxacin, TET—tetracycline, VAN—vancomycin, CSF—cerebrospinal fluid, n.s.—not specified

**Fig 1:**
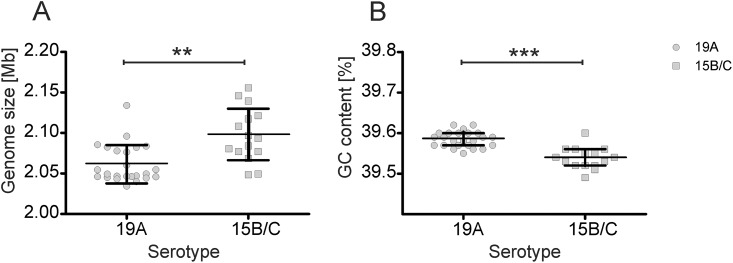
Distribution of the genome sizes (A) and the GC contents (B) of the analysed ST199 isolates with serotype 19A or serogroup 15B/C. Significance is indicated by asterisks (** P < 0.0013, *** P < 0.0001).

Because the serotypes 15B and 15C only differ in mutations in the *wciZ* gene of the *cps* locus, their genome data sets were merged, and the estimated mean of the serogroup 15B/C isolates was 2.098±0.033 MB. As presumed, the genome sizes of serotypes 15B and 15C did not differ significantly (p = 0.9839): In contrast to serogroup B/C (P = 0.6735), the genomes of the serotype 19A did not pass the normality test (P = 0.0022), and the stronger dispersion of the values indicated higher sequence variability within this serotype. The mean genome size of the serotype 19A isolates was 2.061 ± 0.023 MB and, despite the observed dispersion, was significantly smaller (P = 0.0007) than the mean genome size of serogroup 15B/C (Fig 1). Significant differences (P < 0.0001) in the GC content were observed between isolates of serotype 19A (39.59 ± 0.02%) and serogroup 15B/C (39.54 ± 0.02%). The GC content in the serogroups was normally distributed (P > 0.5). However, these data were obtained on scaffolds and must therefore be treated with caution.

No plasmids were identified, but one to three prophages or related signatures were observed within the assembled genomes of all 39 isolates. In two serotype 15B isolates (>32,500 bp in isolate ID 41844 and >37,500 bp in isolate ID 13827), two scaffolds could not be unambiguously assembled into the chromosomes. A BLAST analysis revealed the phage-related origins of these scaffolds. In isolate ID 13827, the unknown scaffold was nearly identical to phage 11865 (FR671409.1) (99%, e-value 0) and phage 23782 (FR671408.1) (98%, e-value 0), both of which have been detected in various *S*. *pneumoniae* isolates with different serotypes. The unknown scaffold of isolate ID 41844 was nearly identical to parts of two other pneumococcal isolates with serotype 11 A (CP002121.1) (99%, e-value 0) and serotype 19A (CP000936.1) (99%, e-value 0).

The pan genome of these isolates consisted of 2,678 genes, with 888 genes that were not identified in all isolates. Among those genes, 241 (27%) were detected in serotype 19A and 280 (32%) were detected in serogroup 15B/C only. Thus, the core genome of the analysed isolates was estimated to consist of 1,790 genes.

### The *cps* locus

**Fig 2:**
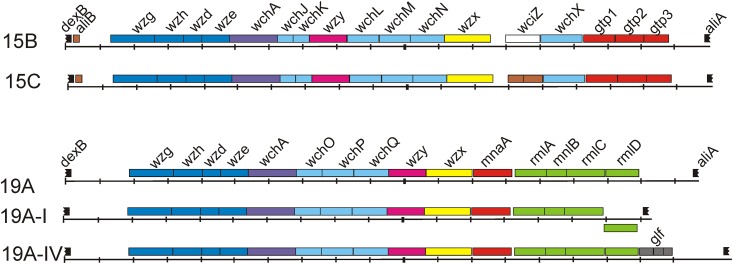
The *cps* clusters of serotypes 15B and 15C and the variations in the *cps* clusters of serotype 19A detected in this study. The genes are coloured using the scheme reported by Bentley et al. [21]: regulatory genes in dark blue, initial transferase in violet, glycosyl transferase in light blue, polymerase in pink, flippase in yellow, acetyl transferase in white, dTDP-L-rhamnose pathway genes in green, UDP-N-acetyl-D-mannosamine pathway genes in red, *glf* genes in grey, pseudogenes in brown, and flanking genes in black. The flanking transposons are not indicated.

The *cps* cluster was approximately 20 kb and was located between the *dexB* and *aliA* region as previously described [21] (Fig 2). As expected, 8 genes were specific for serotype 19A and 10 genes were specific for serogroup 15B/C. The first four conserved genes, *wzg*, *wzh*, *wzd* and *wze*, which encode regulatory proteins, and the *wchA* gene, which encodes the initial transferase, were detected in all analysed strains.

In all strains of serogroup 15B/C, 12 genes downstream of *wchA* were identified that were conserved among the analysed isolates and identical to those annotated by Bentley *et al* [21]: *wchJ*, *wchK*, *wzy*, *wchL*, *wchM*, *wchN*, *wzx*, *wciZ* (2-bp frameshift of the TA tandem repeats in serotype 15C), *wchX*, *gtp1*, *gtp2*, and *gtp3*.

Downstream of *wchA*, strains of serotype 19A bear 10 genes organized in a serotype-specific manner: *wchO*, *wchP*, *wchQ*, *wzy*, *wzx*, *mnaA*, *rmlA*, *rmlB*, *rmlC* and *rmlD*. However, differences in gene composition were observed at the 5’-end compared to the annotation of Bentley *et al* [21], who sub-grouped the serotype 19A isolates into two *cps* variants. In one group (n = 8), the *rmlD* gene was divergently oriented and has been described as serotype 19A-I [22], which indicates that within serotype-specific *cps* clusters variations in the genes and their positions can occur without notable phenotypic changes. The other serotype 19A cluster (n = 17) was named 19A-IV and contained the *rmlD* gene, which was oriented as described by Bentley *et al* [21], but the *glf* pseudogenes with three overlapping open reading frames (ORFs) were also identified downstream of *rmlD*. In general, the *glf* pseudogenes were observed in different arrangements within the known *cps* clusters, mainly upstream of *rmlD* or within the central region upstream of *ugd* or *wciG* or *wzy*. A BLAST analysis revealed a similar arrangement of the *glf* gene in the serotype 19A strain TCH8431 isolated from the respiratory tract that was sequenced during the human microbiome project (NCBI accession number CP001993). The functional *glf* gene encodes UDP-galactopyranose mutase, a 387-amino acid protein involved in the conversion of UDP-α-galactose to UDP-α-galactofuranose and has been experimentally identified in some mycobacteria and Gram-negative species [23, 24]. The *glf* ORFs in the *cps* cluster encode three short putative proteins predicted for various serotype *cps* clusters, but the function of the pseudogenes remains elusive. Thus, the sequence variations that caused ORF rearrangements led to a loss of initial *glf* function. A short, 114-bp sequence was located directly downstream of the *glf* gene and was spread throughout some annotated *S*. *pneumoniae* genomes of various serotypes (6A, 6B, 7A, 7B, 7F, 13, 18B, 18C, 19A, 24A, 24F, 25A, 29, 33D, 38, 40), including clone TCH8431 and the PMEN clone Taiwan^19F-14^. This sequence is often located upstream or downstream of putative ORFs, and genes with known functions, such as the *fatB* gene that encodes a siderophore binding protein, the *prsW* gene that encodes a membrane proteinase of anti-sigma factor RsiW, certain *gcnA*-like genes that encode N-acetyl-β-D-glucosaminidases, a gene encoding the IIB subunits of the PTS, and the *clpA* gene that encodes the ClpX protease. This short sequence is also located upstream of *aliA* in various *cps* loci when the *glf* is missing [21], which suggests that the *glf* locus became a part of the recombination system and might be involved in rearranging the *cps* cluster.

### Phylogenetic analysis and serotype switching

**Fig 3:**
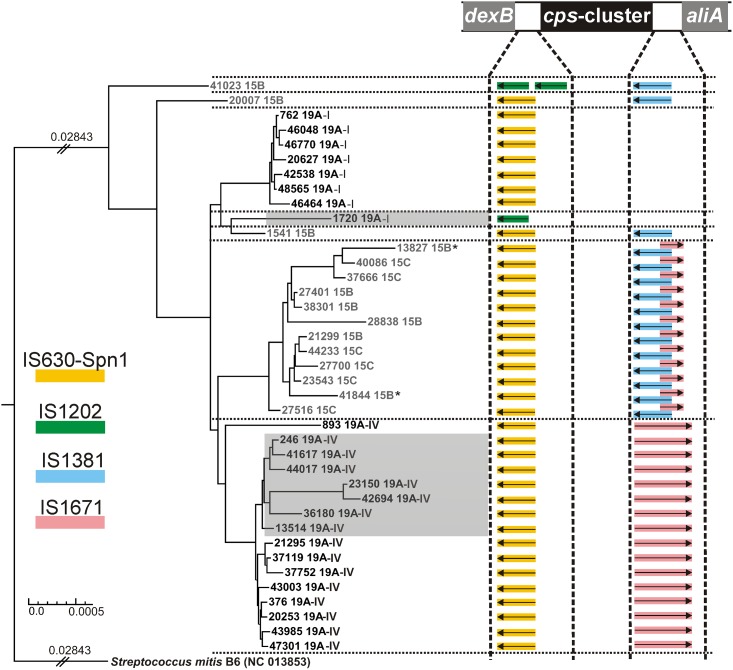
Phylogenetic relationship based on the core genome of 1388 genes derived from the 39 *S*. *pneumoniae* isolates with ST199 and *Streptococcus mitis* (as outgroup). The two isolates with phage-specific sequences that could not be assembled into the chromosomes are indicated by asterisks. The low-level penicillin-resistant isolates are underlined in grey. The transposases (*tnp*) flanking the *cps* clusters are shown as coloured arrows, and the legend for the *tpn* sources is indicated on the left side. The different constellations of *tpn* genes correlated with the linages are separated by dotted lines.

We performed a phylogenetic analysis to identify any serotype replacement within the 39 isolates of ST199 using *Streptococcus mitis* NC 013853 as the outgroup. In this manner, the phylogeny was estimated based on 1388 overlapping genes. Seven distinct clades could be identified, with 3 belonging to serotype 19A and 4 belonging to serogroup 15B/C (Fig 3). The analysis of the DNA sequences of the regions flanking the *cps* cluster (up to 5’ *pbp2X* upstream to 3’ *pbp1a* downstream) revealed differences in the transposase gene (*tnp*) pattern within the clades, suggesting that recombination events had occurred.

The serotype 19A isolates showed two distant clades that corresponded to the variations identified in their *cps* clusters. The major clade 19A-IV contained 15 isolates and was related to isolate ID 893, which showed some phylogenetic distance to the other 19A-IV isolates. All serotype 19A-IV isolates carried the *tnp* of IS630-Spn1 upstream of *wzg* and *tnp* of IS1671 downstream of *rmlD*, as previously described [21]. The serotype 19A-I clade carries the *tnp* of IS630-Spn1 upstream of *wzg*, but the expected *tnp* of IS1671 downstream of *rmlD* was likely lost during the inversion process of the *rmlD* gene.

Two serotype 15B isolates (ID 41023 and ID 20007) represented two distinct clades, and their *cps* clusters were flanked by different *tnp* gene patterns. Both isolates contained a *tnp* gene of IS1381 downstream of *gtp3*, but two copies of the IS1202 *tnp* were identified upstream of *wzg* in ID 41023, which showed the longest distance to the other isolates, whereas isolate ID 20007 was flanked by the *tnp* of IS630-Spn1.

The 19A-I isolate ID 1720 showed an uncommon *tnp* pattern, with missing transposase structures downstream of the inverted *rmlD* gene but one IS1202 copy upstream of *wzg*, indicating some cross-interactions with the ID 41023 linage.

The major clade of serogroup 15B/C was more closely related to the serotype 19A-IV than it was to the two distant serotype 15B isolates, indicating a serotype switch and further diversification of both clades. All 15B/C isolates of this clade contained the *tnp* gene of IS630-Spn1 upstream of *wzg* that was common in all 19A and 15B/C serotypes. However, the *tnp* of IS1381 that was also present in the distinct serotype 15B isolates ID 41023 and ID 20007 was located downstream of *gtp3*, followed by an artefact of IS1671, the *tnp* common in 19A-IV. Considering the nature of the recombination process this *tnp* pattern supports the idea of a 19A-IV ancestor. The 15B/C clade contained 6 isolates of serotype 15B and 5 isolates of serotype 15C distributed throughout the lineages. The difference between both serotypes is a 2-bp insertion (AT) within a poly-(AT)-region. It has been shown that tandem repeats exhibit a highly increased rate of frameshift mutations due to deletions or insertions [25], which suggests that serotype 15C emerged *via* mutations rather than recombination processes.

A second serotype switch was observed for serotype 15B isolate ID 1541, which showed a genetic relation to serotype 19A-I isolate ID 1720. Because of their distances to each other and the other 19A-1 isolates, both are suggested to belong to two separate clades. Both regions flanking the *cps* of ID 1541 were similar to ID 20007 *cps* regions. ID 1541 shared identical point mutations in the *wzg*, *wzh* and *wze* genes with isolate ID 20007, indicating that the *cps* cluster recombined between the ancestors of these isolates. The different *tnp* pattern of serotype 19A-I isolate ID 1720 that showed only a IS1202-like sequence identified upstream of the *cps* locus of the serotype 15B isolate ID 41023 might also indicate a serotype switch, but it is difficult to interpret the event because no closer relatives of this isolate were sequenced in this study.

### Resistance to antimicrobials

Serotype 19A is associated with antibiotic resistance; therefore, the phenotypic resistance profiles and possible genetic determinants for the most recommended antimicrobials for treating CAP (Table 1) were investigated: macrolides (clarithromycin), fluoroquinolones (levofloxacin) and β-lactams (penicillin and cefotaxime). Susceptibility to alternative antimicrobials, such as clindamycin, tetracycline, and vancomycin, was also investigated.

The isolates were susceptible to most of the antibiotics tested, except for certain isolates with resistance to clarithromycin (minimum inhibitory concentrations (MIC) > 0.5 mg/L). Here, one 15B (ID 28838) and two 19A-IV isolates (ID 23150 and ID 42694) showed a MIC of 4 mg/L clarithromycin. This macrolide resistance could be traced to the transposon-associated *mefA* and *msrD* genes that encode dual macrolide efflux protein A (MefA and Mel, respectively) [26] (100% identity, e-value 0).

Two isolates of serotype 19A-IV (ID 37119) and 19A-I (ID 42538) showed intermediate MICs of levofloxacin of 2 mg/L each. The increased fluoroquinolone MICs might be caused by substitutions in the quinolone resistance-determining region (QRDR) of the subunits of topoisomerase IV (ParE/ParC) and/or gyrase (GyrA/GyrB) [27] or by the increased expression of multidrug efflux pumps, primarily PmrA [28] and PatAB [29]. Substitutions were identified outside the QRDRs in the ORFs of ParC (A382T and A596V) and ParE (V162I, P165L, S216) and within the QRDR of ParE (I460V) in both isolates. However, these substitutions were also identified in other levofloxacin-susceptible isolates [30]; therefore, it remains unlikely that these substitutions outside the QRDR and the I460V substitution within the QRDR are associated with the increased MIC of levofloxacin. No differences were observed in the *pmrA* promoters [28] or the *patAB* terminator regions in the resistant isolates that might lead to enhanced expression or attenuation [31]. However, the transcriptional levels of the genes encoding the efflux pumps were not quantified. Moreover, it was suggested that other unknown efflux pumps might be involved in the drug resistance in pneumococci [32].

All isolates were susceptible to the cephalosporin cefotaxime, but low-level penicillin resistance (MICs > 0.06 to 0.25 mg/L) was observed in six isolates (ID 13514, ID 23150, ID 36180, ID 41617, ID 44017 and ID 42694). Those formed their own sub-linage within clade 19A-IV (labelled as 19A-IV’ in Fig 3), of which only isolate ID 246 showed the MIC of penicillin of 0.06 mg/L. According to the European Committee on Antimicrobial Susceptibility Testing (EUCAST) and based on the epidemiological cut-off (ECOFF) that separates the natural susceptible population, the MIC of penicillin > 0.06 mg/L) is interpreted as nonsusceptible and as resistant when meningitis is the focus of infection (http://www.eucast.org/clinical_breakpoints/). The main mechanism of high-level resistance (MIC > 4 mg/L) to β-lactams is related to amino acid substitutions in the active site (motifs SXXK, HSXN and K[ST]G) of penicillin binding proteins (PBPs), whereas low-level resistance is related to various other substitutions within PBPs, mainly in PBP1A, PBP2B and PBP2X [33, 34], and other proteins of peptidoglycan synthesis [35]. The *cps* cluster is proximally flanked by the genes *pbp2x* (8 kbp upstream) and *pbp1a* (7 kbp downstream), which have been shown to be involved in the recombination processes of the *cps* cluster and thus might lead to the transmission of penicillin resistance *via* serotype switching [36]. However, penicillin resistance might act as the driver and the switch might be the result of the gain in resistance.

To investigate whether the observed low-level resistance was caused by already described or new substitutions, translated ORFs of the penicillin binding proteins (PBPs) and other genes associated with increased MICs of β-lactams of the nonsusceptible isolates were compared to the other susceptible isolates.

**Fig 4:**
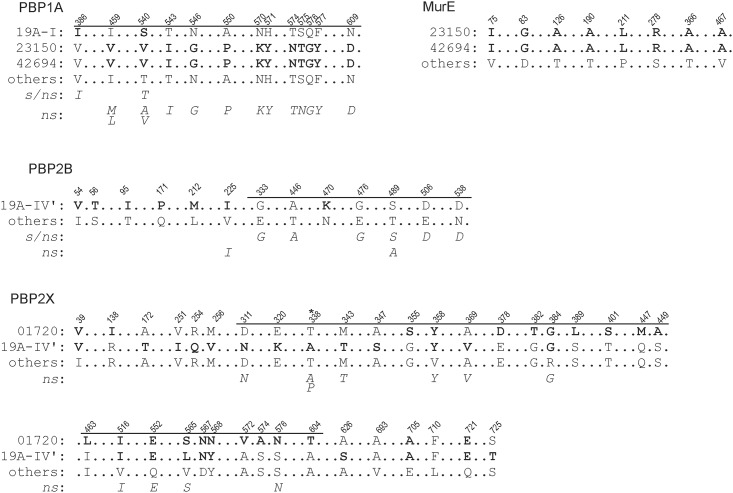
Amino acid substitutions identified in the PBPs (1A, 2B and 2X) and MurE of the ST199 isolates in this study (bold letters) and of other isolates published elsewhere (italic letters). ‘Others’ indicates the protein sequence of all merged isolates of this study, except for those indicated by their ID. The nonsusceptibility bases on the epidemiological cut-off (ECOFF) > 0.06 mg/L that indicates the breakpoint between the susceptible (*s*) and nonsusceptible isolates (*ns*). The position of the conserved motif SXXK of the PBPs is indicated by asterisks. The amino acid positions, as well as the TP-domain of the respective PBPs shown as bars, are indicated above the sequences. Sequences published elsewhere (italic letters) refer to [35, 37, 41–44].

Isolates belonging to the sub-lineage of clade 19A-IV’ showed the same amino acid substitutions within PBP2X and PBP2B, the class B monofunctional transpeptidases, and the corresponding gene sequences were identical, which indicated that these mutations were specific for this sub-lineage of 19A-IV. Some substitutions had not been previously described but were located within the N-terminally located non-penicillin-binding transmembrane domain (n-PB, up to residue 265) or the C-terminal domain (residues 635–750) [37, 38]. The substitutions within the n-TP-domain likely do not influence the phenotypes of either PBP2B or PBP2X. The C-terminal domain contains two PASTA (penicillin-binding protein and Ser/Thr kinase associated) motifs and is suggested to bind to uncross-linked murein; it was also shown to interact with a second β-lactam. The impact of the mutations found in this domain on PBP2X is unknown. Most of the identified substitutions within the transpeptidase domain (TP, residues 266 to 616) of PBP2X [37, 38] were previously identified in other nonsusceptible isolates (italic letters in Fig 4) [37, 39, 40]. Only three substitutions within the TP domain of PBP2X, E320K, A347S, and D567N were not described (leucine (L) at position 565 was found in the susceptible strain R6). Asparagine (N) at 567 was also identified in the susceptible isolate ID 1720, thus this mutation does not appear to be critical. However, substitutions of the negatively charged glutamic acid (E) to positively charged lysine (K) at position 320 and from hydrophobic alanine (A) to hydrophilic serine (S) at position 347, both closely located to the S^337^TMK (SXXK) motif, might have an impact on the penicillin nonsusceptibility. Interestingly, we found four substitutions within the TP domain of BPB2X, being related to nonsusceptible strains, also in isolate ID 1720 (V358Y, R384G, Q552E, and S576N) as well as some unknown mutations (Fig 3) that seem to be specific for this strain or lineage and suggest that these mutation might be not crucial for the phenotype.

All substitutions found within the TP domain of the PBP2B in the sub-lineage 19A-IV’ have been previously described, primarily in nonsusceptible isolates (except for one susceptible isolate indicated by *s/ns* in Fig 4 for PBP2B). Therefore, it is likely that those mutations lead to an increased penicillin MIC. The susceptible phenotype of isolate ID 246 suggested additional alterations in other unknown genes that were not further investigated.

The isolates ID 23150 and ID 42694, both of which are located in a distant clade but are strongly related to each other, showed a similar substitution pattern outside the conserved motifs of the TP-domain (residues 337 to 681) of PBP1A [45] (Fig 4), the class A bifunctional transglycosylase-transpeptidases. All of these substitutions have already been described for intermediate and resistant isolates [41] and mutagenized clones [42], and thus might contribute to the increased MIC of penicillin in these isolates. Both isolates also exhibited identical substitutions within MurE, a UDP-N-acetylmuramyl tripeptide synthetase. However, in the case of MurE, mutations in the promoter region leading to increased transcription levels have been shown to increase β-lactam resistance in Gram-positive species, particularly resistance to oxacillin [43, 46]. Thus, it remains unclear whether those substitutions have any impact on the phenotype. Interestingly, isolate ID 42694 showed the highest MIC of penicillin that might be caused by these observed substitutions. However, because isolate ID 23150 showed a lower MIC, it cannot be excluded that unknown mechanism might be involved either in the increased MIC of ID 42694 or the lower MIC of ID 23150.

The isolate ID 1720 showed also two substitutions within PBP1A (V386I and T540S) and in PBP2A (A73V and A172G) with unknown effects. No amino acid changes in PBP1B and PBP3 or in non-PBP-targets (CpoA, CiaH, CiaR, MurN or MurM) that were associated with the increased MIC of β-lactams [35] were identified within the tested isolates.

## Conclusions

The number of multidrug resistant pneumococcal clones, including those with penicillin resistance, was successfully reduced by the PCV7 [47–49] and the PCV13 vaccine programme for infants [50, 51]. Before 1987, the rate of penicillin nonsusceptible isolates (MIC of ≥ 0.1 mg/L) associated with IPD has been estimated at 5% in the USA, but increased to 25.1% in 1999, with PCV7 serotypes accounting for ∼80% of the cases [51]. These could be reduced to 17% by PCV7 and 14.4% by PCV13 in USA [50]. In some European countries, such as France (56.7%), Greece (36.4%) or Spain (54.5%), the prevalence of penicillin nonsusceptible isolates was greater before the introduction of the PCV7 in Europe (2001) [52]. After the introduction of PCV7, the incidence of non-PCV7 serotypes increased, and 19A was among the most frequent serotypes that were also associated with increased penicillin resistance [12, 36]. In this study, six isolates of serotype 19A, that could be phylogenetically sub-grouped to the sub-linage 19A-IV’, showed increased penicillin MIC values and were categorized as possessing low-level penicillin resistance. The sub-linage 19A-IV’ shared identical substitutions in the PBP2B and PBP2X. Isolate ID 13514 was obtained in 1999; therefore, these substitutions appear to have been established before the PCV7 vaccine was introduced. Interestingly, isolate ID 246 was isolated in 1998 and was susceptible to penicillin but bears the same substitution pattern in PBP2B and PBP2X. Thus, the low-level penicillin resistance in this sub-lineage might also be caused by other unknown mutations that spread clonally. In two strains that were isolated in 2005 (ID 23150) and in 2010 (ID 42694), the pattern of substitutions was extended to PBP1A and MurE, but the relevance of these substitutions to the increased MIC of penicillin in isolate ID 42694 remains unclear. The most recent clone, ID 42694, might have established additional unknown mutations in other targets, but these additional mutations were not analysed in this study.

An analysis of the serotype 19A isolates collected by the CDC Active Bacterial Core Surveillance programme in the USA revealed that the vast majority of the isolates could be assigned to 3 major clonal complexes: CC 199, CC3 20/271, and CC 695. These 3 clonal complexes collectively comprised approximately 87% of the serotype 19A isolates collected from 2005–2007 [53]. CC 199 was the most prevalent clonal complex of serotype 19A before the PCV7 vaccine, and the IPD caused by serotype 19A of CC 199 declined gradually from 72% (in 2003–2004) to 40% (in 2007) in the USA, indicating changes in the epidemiology of serotype 19A [54]. Simultaneously, IPDs caused by serotype 19A with increased penicillin MIC values belonging to CC 320/271 and CC 695 increased in USA. Before the introduction of PCV7, these clonal complexes were primary related to serotypes 19F and 4. Both serotypes are covered by PCV7, and their prevalence was strongly reduced between 1998/1999 and 2006/2007 from 11.1% to 1.2% (data for serotype 19F) and 6.8% to 0.2% (data for serotype 4). Because penicillin resistance in pneumococci is often associated with multi-drug resistant phenotype, it was suggested that the observed serotype switches of those clonal complexes to 19A were driven by selective antimicrobial pressure [54]. A transmission and recombination of the *pbp2x*-*cps* locus-*pbp1a* from CC 199^19A^ (donor) to CC695^4^ (recipient) was proposed to result in CC695^19A^ [36]. The *pbp2x* genes of the nonsusceptible strains of this study were also identical to the serotype CC 199^19A^ donor strain cdc2 described by Bruegemann et al. [36]. In Germany, other than CC 199 (30.4%), the most prevalent clonal complex related to 19A was CC 230 (18.4%) until 2011 [15]. Before the introduction of PCV7, intermediate penicillin resistance was only observed in CC 230 [15], which indicated strong regional differences in the epidemiology of the clonal complexes.

In the present study, at least two serotype switches from 19A to 15B of ST199 belonging to CC 199 were identified. Based on the IS pattern flanking the *cps*-cluster, one of the switches was most likely from serotype 19A-IV to serogroup 15B/C. It remains unclear when the serotype switch occurred because almost all of the isolates were obtained after 2004, but one serotype 15B strain (ID 13827) was isolated in 1999; thus, this serotype switch may have occurred in or before 1999. The second serotype switch from 19A-I to 15B is more difficult to interpret because closer relatives are missing but must have occurred before 2003. Because both serotype switches occurred before the introduction of PCV13 in Germany in 2009, the vaccination did not directly provoke the serotype switch. Some ST199 isolates within serogroup 15B/C were already identified in the pre-PCV era, suggesting that the recombination of the *cps*-cluster from 19A to 15B among ST199 isolates is not unusual. Temporal changes in pneumococcal epidemiology already occurred before the introduction of the conjugated vaccines [55] and cannot always be sufficiently explained. It can be assumed that recombinations occur at random and that recombinants with advantageous capsules are selected. However, the likelihood for recombination with pneumococci bearing a non-vaccine serotype also increases with their prevalence. Therefore, the passive selective pressure of the vaccine not only selects recombinants with non-vaccine serotypes but might also increase the likelihood of such recombination.

This study shows that there is a dynamic flexibility in CC 199 population genomics. In particular, the capsular switch between serotypes 19A and serogroup 15B/C appears to be a rather frequent event and had already occurred in isolates collected before the PCV7 selective pressure. Therefore, it can be assumed that in response to the selective pressure from PCV13, which covers serotype 19A, the low hurdles for this *cps* recombination event will trigger a vaccine escape towards serogroup 15B/C due to the already existing serotype-switched clones that can successfully occupy the vacant niche. The switched serotype 15B isolates were penicillin susceptible, but as our results indicate, a serotype recombination with the penicillin nonsusceptible sub-lineage 19A-IV’ is highly possible. Indeed, there are recent reports describing an increased detection of serogroup 15B/C in IPD including those isolates with MIC of penicillin >0.6 mg/L [20, 56]. This issue is concerning because the current development of an extended PCV with 15 serotypes will not cover serogroup 15B/C.

In conclusion, the PCVs displaced the most prevalent strains, revealing the highly dynamic serotype changes that can lead to the successful spread of escaping serotypes. Those changes can only be reliably identified by NGS and may help predict the most probable trends in pneumococcal epidemiology. These techniques and studies should also be considered by retrospective epidemiological studies performed by the pharmaceutical industry when strategic decisions regarding the composition of a vaccine are made before beginning the resource- and time-consuming clinical development.

## Materials and Methods

### Bacterial strains and culture

The *Streptococcus pneumoniae* isolates were collected in Germany from 1998 to 2011 in various studies by the German National Reference Center for Streptococci (Aachen, Germany). The characterization of the sequence type (ST) was performed using multi locus sequence typing (MLST), and serotyping was performed using the Neufeld Quellung reaction, as previously described [15]. Antibiotic susceptibility was tested according to the recommendations of the European Committee on Antimicrobial Susceptibility Testing (EUCAST) by the double-dilution method. In total, 39 isolates of ST199, consisting of 24 isolates of serotype 19A, 9 isolates of serotype 15B and 6 isolates of serotype 15C, were included in this study (for details, see Table 1).

The *S*. *pneumoniae* strains were inoculated into 10 ml Todd-Hewitt broth and grown for 8 to 10 hours at 35°C in a 5% CO_2_ atmosphere in 50 ml tubes. The cells were harvested by centrifugation at 4,000 rpm.

### DNA preparation

The bacterial pellets were washed in buffer A (20 mM Tris-HCl (pH 8.0), 50 mM NaCl, and 10 mM EDTA (ethylenediaminetetraacetic acid)) and resuspended in 700 μl of buffer A supplemented with 4 mg/mL lysozyme. The cell suspension was chilled on ice for 10 min, followed by a 20-min incubation at 37°C. The cells were lysed by adding 25 μL of N-laurylsarcosyl (30%) and 10 μL of proteinase K (20 mg/mL) and were incubated at 70°C for 20 min. Chromosomal DNA was purified by three sequential extractions with a Tris-EDTA-saturated phenol/chloroform/isoamyl alcohol (25:24:1) mixture (Roth GmbH, Germany). The inorganic phase was separated by centrifugation at 10,000 rpm for 5 min and was treated with 5 μL of RNAse (100 mg/ml) for 15 min at 37°C. The DNA was precipitated for 10 min on ice by adding of 3 volumes of ice-cold ethanol (96%), and then pelleted by centrifugation at 13,000 rpm for 15 min at 4°C. The pellet was washed once with 300 μl of ice-cold ethanol (70%) and air-dried. The DNA was resuspended in DNase-free water (Gibco, Thermo Fisher Scientific, Waltham, MA USA) overnight and stored at 4°C.

### Genome sequencing and assembly

For each sample, a whole-genome sequence library was prepared using the Illumina-Compatible Nextera DNA Sample Prep Kit (Epicentre, Madison, WI USA), according to the manufacturer's protocol. Each library was tagged with an individual tag combination and a library pool containing equimolar amounts of the individual libraries was prepared. The library pool was sequenced in 2x250 bp paired read runs on the MiSeq platform, yielding 21,928,122 total reads. After de-multiplexing, the individual sample reads were assembled using the Newbler assembler v2.8 (Roche, Branford, CT USA). Contigs of the initial Newbler assemblies (unordered drafts) were then aligned to the reference genome of *S*. *pneumoniae* ATCC 700669 using MUMmer [57], and ordered drafts were constructed using a combination of *ad hoc* Perl and shell scripts. Contigs that could not be assigned to a position were placed in separate FASTA files.

### Gap closure of the *cps* locus

Gap closure of the flanking regions of the *cps* locus was performed by Sanger-sequencing. Primers that bound within *dexA* (GTTCCATGGGATGCTTTCTG) and *wzg* (TCGCTTCACTTTCTGTGAAC), and within *aliA* (AATAATGTCACGCCCGCAAG) and *wciA* (serogroup 15B/C) (AGGAGAAGCAACGGTGAATG-3) or *glf* (serotype 19A) (TGAGTTTGGGAGTCAAGCAAAG) were designed and purchased from Metabion GmbH (Germany) to amplify the regions. The PCR was performed in a 20 μl reaction using 3 ng of chromosomal DNA, 0.2 μM primers, 200 μM dNTPs, and 0.4 U of Phusion^™^ High-Fidelity DNA Polymerase in 1 x Phusion^™^ HF Buffers (New England BioLabs Inc., Ipswich, MA USA). The following PCR algorithm was applied: 98°C for 30 s; 29 cycles of 98°C for 10 s, 62°C for 20 s, and 72°C for 30 s; and 72°C for 10 min. The PCR products were extracted from agarose gels using the QIAquick Gel Extraction Kit (Qiagen GmbH, Hilden, Germany) or by ExoSAP-IT^™^ (Affymetrix Inc, Santa Clara, CA USA.), according to the manufacturers’ protocols. Sanger sequencing of the PCR products was performed on a 3730xl DNA-Analyzer (Applied Biosystems, Thermo Fisher Scientific, Waltham, MA USA) by IIT Biotech GmbH using the capillary sequencing technique.

### Genome annotation

The genomes were annotated using the platform GenDB [58]. The contigs of each draft sequence were concatenated into one contig using a 6-frame stop linker (ctagcatgctag), which was then annotated using the gene and function prediction pipelines in GenDB. If unassigned contigs were present in a genome, these contigs were concatenated separately and uploaded as an additional contig. The MLST strain types were confirmed by analysing the respective alleles using MLST 1.7 [59]. Phage-specific sequences were determined using PHAST [60]. Genes encoding resistance determinants were identified by ResFinder 2.1 [61], and the amino acid sequences were compared using MUSCLE 3.8 [62] and further analysed by sequence alignments using CLC Main Workbench (Qiagen).

### Phylogenetic analysis

The calculation of the core genomes and construction of the phylogenetic tree was performed with the software tool EDGAR [63]. *Streptococcus mitis* B6 [64] was used as an outgroup to root the tree. First, the core genes of the 40 genomes were computed. In the next step, multiple alignments of the 1,388 core genes were generated using MUSCLE [65]. The alignments were than concatenated into a large multiple alignment, which was subsequently used as input for PHYLIP [66]. Here, a distance matrix based on the Jones-Taylor-Thornton model [67] was calculated from this alignment, and finally, based on this matrix, a phylogenetic tree was constructed using the Neighbour-Joining method. The tree was visualized by the program TreeGraph 2 [68]. The draft genomes of the 39 isolates were aligned with progressive Mauve [69] using standard parameters and a scoring matrix.

### Statistical analysis

All statistics were performed using GraphPad Prism version 6.00 for Windows (GraphPad Software, La Jolla California USA, www.graphpad.com.) The distributions of the genome sizes and the GC contents were analysed using the D'Agostino & Pearson omnibus normality test and the differences were determined using unpaired t tests with Welch's correction (normal distribution) or Mann-Whitney test (non-parametric). Significant differences were assumed as P-value of ≤ 0.05.

### Ethical statement

Ethical approval was not required because the study did not involve human subjects, material or data.
